# The growth and development of cress (*Lepidium sativum*) affected by blue and red light

**DOI:** 10.1016/j.heliyon.2019.e02109

**Published:** 2019-07-27

**Authors:** Ladan Ajdanian, Mehdi Babaei, Hossein Aroiee

**Affiliations:** a Department of Horticultural Sciences, Faculty of Agriculture, Ferdowsi University of Mashhad, Mashhad, Iran; b Department of Horticultural Sciences, Faculty of Agriculture and Natural Resources, University of Tehran, Tehran, Iran

**Keywords:** Agriculture, Cryptochrome, LED lamp, Phytochrome, Anthocyanin

## Abstract

Today, the use of light emitting diodes (LEDs) are rapidly increasing in horticulture industry as a result of technological advancements. Lighting systems play an important role in the commercial greenhouse productions. As an artificial source of light, LED lamps can contribute to the better and faster growth of horticulture products such as vegetables grown in greenhouses. In this study, the effects of red and blue light spectrums were implemented and performed as a pot experiment under the cultivation-without-soil condition in greenhouse based on a completely random plan with 3 lighting treatments including natural light (control), 60% red light +40% blue light (60R:40B), and 90% red light +10% blue light (90R:10B), repeated 3 times. The results showed that the application of blue and red lights affected the fresh and dry weights of cress as well as its biomass, demonstrating a considerable increase compared to the plants grown under natural sunlight condition. In this regard, the fresh weight of the plant under the 60R:40B treatment had 57.11% increase compared to the natural light treatment. Compared to the control sample, the dry weight had 26.06% increase under 90R:10B treatment. The highest extent of biomass was observed under the 60R:40B lighting treatment with a value of 1.51 (g per kg dry weight of the plant), which was a 68.87% increase compared to the natural light treatment. Under the 60R:40B treatment, cress had its highest length at 19.76 cm. Under the similar treatment, the cress leaf had a total area of 56.78 cm^2^ which was the largest. The stem diameter and the number of leaves under the 60R:40B treatment had their highest values at 3.28 mm and 8.16, respectively. Accordingly, a growing trend was observed with 56.7 and 61.27% increase compared to the control treatment. Furthermore, the biochemical traits of cress, the amount of a, b and total chlorophyll, the amount of anthocyanin and phenolic contents under the application of red and blue light were at their highest values compared to the control treatment. The highest amount of chlorophyll was observed under 60R:40B treatment as 15.09 mg g^−1^ FW leaf. Moreover, the phenolic contents and the amount of anthocyanin were of significant difference at 1% level of likelihood compared to the control treatment. Therefore, the vegetative growth of cress was substantially affected by red and blue lights, resulting in the enhancement of the plant's biochemical features compared to control condition via adjusting the lighting quality and impacts of each red and blue light spectrum on their specific receptors. As a result, the presence of both lighting spectrums is essential for expanding and increasing the quality of the plant. At the large scale, this technology is capable of improving the commercial greenhouse production performance while helping farmers achieve maximum products. This particular combination of lights is one of the beneficial features of LED lighting systems intended for different types of commercial greenhouse productions, especially the valuable greenhouse products.

## 1 Introduction

Light is not only a necessary source of energy for plants, but also an important signal which plays a major role in plant growth, morphological characteristics, cell molecule biosynthesis, and gene expression during the entire growth period of plants. Each different light spectrum has an exclusive effect on particular gene expressions in plants resulting in a number of different impacts (Huché-Thélier et al., 2016). Several processes such as photosynthesis, germination, flowering, and biomass accumulation can be controlled and optimized via adjusting light wavelengths (Pinho, 2008; Yeh and Chung, 2009; Vänninen et al., 2010). Since plants are stationary creatures, they are in a constant competition over gaining light, space, water, and nutrients. Competition over the reception of light leads to morphological and growth-related changes in plants (Pinho, 2008; Yeh and Chung, 2009; Vänninen et al., 2010). Plants have the ability of detecting small changes in light spectrum, intensity and direction. Light receptors sense these signals which provide access to the information used for plant growth regulation. Known light receptors are classified into three major groups including phytochromes, sensitive to red light and far red; cryptochromes, sensitive to UV-A and blue light (Cashmore et al., 1999); and phototropins (Briggs and Huala, 1999). Accordingly, recent studies have shown that the most important wavelengths for photosynthesis are blue and red light wavelengths, and the highest extents of photosynthesis (photosynthetic peak) were observed in 440 nm wavelength (blue) and 620 nm wavelength (red) (Franklin and Whitelam, 2005). Red light plays an important role in developing photosynthesis devices while controlling the changes in light of the phytochrome devices (Urbonavičiūtė et al., 2007). Numerous studies have demonstrated that the use of mixed light spectrums results in the activation of a set of complex light systems in plants which ultimately leads to physiological and biochemical responses from the plants (Ohashi-Kaneko et al., 2006; Huché-Thélier et al., 2016). Using the LED technology, monochrome light and specific spectrums can be produced via combinations of different waves. Today, LEDs with high energy and the ability to produce several wave spectrums are accessible in the horticulture industry (Pinho et al., 2007). Though the manufacturing of LEDs with complete spectrums are more costly and complex, certain LED manufacturers are currently engaged with their production for commercial purposes. Of the advantages of LEDs over regular HPS lights include their longer lifetime and emission of less direct heat towards plants (Morrow, 2008). The low-heat feature in LEDs allows for the implementation of lighting close to the plants, facilitating the exposure among and inside each culture row (Massa et al., 2008). LED lamps have always been considered as low-consumption lamps; however, despite how LEDs' extent of energy consumption is of small difference with HPSs, the technology is rapidly growing in favor of the former (Pinho et al., 2007). Given the research in this area, LEDs produce almost twice the hours of lighting compared to HPS lamps (Pinho et al., 2007). For years, studies were focused on the red spectrum and later, the observable blue spectrums in LED lights (Pinho et al., 2007). Projects such as (Bula et al., 1991) and similar ones at the time were supported by NASA. NASA has also continued their participation in LED related research since 1980 until present. NASA has shown interest in finding a good-quality source of light that involves proper energy consumption and low heat production while being suitable for growing edible plants in irregular conditions (Massa et al., 2008). Studies conducted on the effect of LED light which has recently received attention in greenhouses and centers for growing and breeding plants show the impact of wavelengths and light colors on quantity and quality of plant productions (Olle and Viršile, 2013). Meanwhile, improper application of artificial lights could result in a set of problems such as increased heat, damage to plant tissues in the form of burned leaves, accelerated or delayed flowering, and increased electricity consumption expenses due to high intensity and/or unsuitable presence period of light within the plant's growth atmosphere (Currey and Lopez, 2013). In general, precise controlling of light environment (such as the quality and intensity of light and the lighting period) with respect to the plant's requirements could enhance the performance, quality and production efficiency of the plant. As a result, greenhouses in which LED sources of lights are used are of high potential power to specifically adjust the environment's lighting. Small plants such as leaf vegetables, small roots, and a number of medicinal plants are the most ideal types of plants to culture at greenhouses where LEDs are used. Currently, plant species cultured at greenhouses equipped with LEDs mostly include leaf vegetables such as lettuce and spinach (Gutu et al., 2013; Kang et al., 2013). LED-interlighting products most commonly consist of blue- and red- LED chip combinations, specifically targeted for excitation of the chlorophyll pigments and thus for enhancing photosynthetic activity (Joshi et al., 2019). Nonetheless, additional spectral compositions, including different ratios of blue, red, far-red and white light have been tested for interlighting (Gómez and Mitchell, 2016; Hao et al., 2012; Hernández and Kubota, 2012; Lu et al., 2012). The presence of blue and red lights in lighting combinations in studies on parsley, lettuce, eggplant, and daisies resulted in an increase in height (Heo et al., 2002; Kim et al., 2004; Hirai et al., 2006; Jeong et al., 2014). Blue light leads to chlorophyll biosynthesis, opening of the pores, and increase in the thickness of leaves (Heo et al., 2002; Urbonavičiūtė et al., 2007). A better growth of the strawberry plant was reported under blue LED treatment compared to red, and the combination of red and blue lights (Folta and Childers, 2008). The effect of blue and red light on changing the height of petunia flower is caused by the effect of blue light and excitation of cryptochromes which result in the production of signals that stimulate gibberellin production and stem height; this, in turn, leads to changes in the extent of blue light presence in the environment and its increase or reduction results in alterations in gibberellin excretion and ultimately, changes in height (Fukuda et al., 2016). Blue light was solely responsible for the increase in the height of basil medicinal plant (Glowacka, 2008). Fukuda et al. (2009) and Gautam et al. (2015) suggested that reducing the amount of red light in the environment alone results in delay or prevention of flowering. The effects of red and blue light on flowering is due to their impacts on the performance of phytochrome B pigments and cryptochromes (Fukuda et al., 2009). The ratio of red light to blue is of substantial importance; a ratio of 3 to 1 in red and blue light combination led to an acceptable growth of strawberry plants compared to the sole application of red or blue. Different red and blue LED light spectrums proved effective in increasing the amount of chlorophyll present in strawberry plant and enhanced the fruit's function (Choi et al., 2015). LED light also had a considerable effect in increasing the pure photosynthesis of dracocephalum plant (*Melissa officinalis*) (Frąszczak et al., 2014).

Cress (*Lepidium sativum*) is a small, herbaceous plant with 1 year of age from the Crucifera family, has a height of 50 cm and is rich with minerals and vitamins A and C which are very beneficial for anemia treatment and blood purification. This plant has extraordinary propertied both as an edible leaf vegetable and a medicinal plant.

Given the effects of blue and red light spectrum on plants, particularly their positive impacts on leaf vegetables, the main purpose of this study is to examine and compare different combinations of blue and red lights on morphological and biochemical feature of cress plant as an important leaf vegetable and medicinal plant relative to the control treatment; this is done in order to introduce the most ideal growing condition of cress in terms of lighting ratios of blue and red spectrum combinations and compare the use of this technology with the control treatment in which natural sunlight is used.

## 2 Materials and method

### 2.1 Plant materials and growing conditions

To examine the effects of blue and red lights, the present study was implemented and conducted as a pot experiment inside a greenhouse through a completely random plan with three lighting treatments including natural light (control), 60% red light +40% blue light, and 90% red light +10% blue light; treatments were repeated 3 times at the research greenhouse of the faculty of agriculture, Ferdowsi University of Mashhad, with a latitude of 36° 16″ North and a longitude of 59° 36” East, altitude of 985m from sea level, mean temperature of 15–27 °C and relative humidity of 40–70%. As the temperature inside the greenhouse was recorded by receivers connected to the greenhouse central system, the ceiling windows and/or ventilators would automatically activate in case of temperature rise. Each lighting treatment consisted of 3 pots with 3 repetitions, amounting to a total of 27 pots. 15 cress seeds were planted inside each pot. The mean data of each pot during the growth period was examined via statistical analysis. In this study, plastic pots with a height of 40 cm and diameter of 30cm were used. The cultivation bed for the plant included a mixture of 40% peat moss, 40% coco peat, and 20% pearlite. Plants were watered daily up to 10 mm, and were subsequently fed with Hoagland solution every other day. Measurements were carried out 35 days following the planting and complete growth of the plant.

### 2.2 Lighting treatments

**Fig. 1:**
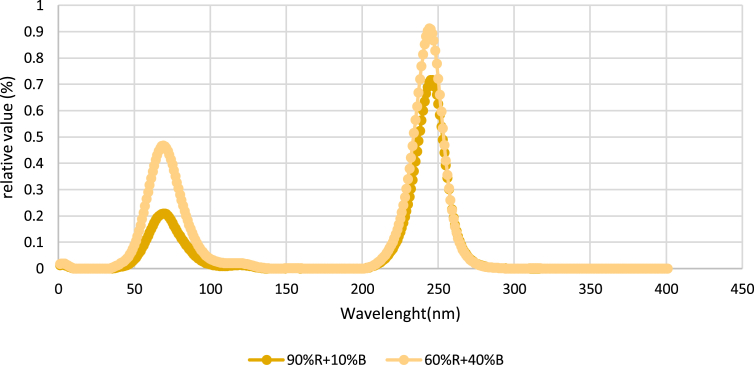
Relative spectral photon flux of the light sources red and blue (RB) utilized.

Plants were illuminated by light emitting diodes (LEDs) with different percentages of red (R, 661 nm) and blue (B, 449 nm) lights. Three spectral treatments were used in this study, namely 90%R+10%B, 60%R+40%B and control. The photoperiod was 12/12h (day/night), photosynthetic photon flux density (PPFD) was 168 ± 10 μmol m^−2^ s^−1^. The LED lights were prototypes from General Electric Lighting Solutions (Salid, Karamax, Iran). These consisted of 0.26 m, 0.06 m, 0.05 m linear fixtures, on which were placed an array of 6 LEDs. Irradiance was measured routinely using a quantum sensor (MQ-200; Apogee Instruments, Logan, UT). Photosynthetic photon flux density intensities and light spectra were monitored using a light meter (Sekonic C-7000, Japan). The relative spectra of the light treatments are shown in Fig. 1.

The distance between lamps and plants were adjustable during different stages of the growth via metal clips. At the control experiment unit, natural sunlight was used. The plants’ growing environment was completely covered using special plastic covers in order to avoid light interference while the lamps were active.

### 2.3 Examined traits

#### 2.3.1 Plant's dry and fresh weights, percentage of dry matter (biomass) in plant's body

To measure the percentage of dry matter, first three samples were provided from each treatment. The fresh weight was measured using a digital scale as 0.01 g. Next, the samples were placed in Oven device at 74 °C for 4 days (until the samples are dried completely); the dry weight of the samples were also measured using a digital scale as 0.01 g ultimately, the following formula was used to measure the percentage of dry matter.

$$\text{Percentage}\;\text{of}\;\text{dry}\;\text{matter}\;=\;\frac{\left(\mathrm{fresh}\;\mathrm{sample}\;\mathrm{weight}-\mathrm{dry}\;\mathrm{sample}\;\mathrm{weight}\right)\;}{\mathrm{fresh}\;\mathrm{sample}\;\mathrm{weight}}\times 100$$

#### 2.3.2 Plant's height

The plant's height was measured and recorded using a tape measure with 0.01 m precision during growth season.

#### 2.3.3 Plant's leaf area

It was measured using Image j software and Leaf Area Meter (Model LI-3100c) device.

#### 2.3.4 Stem diameter

It was measured using a caliper with 0.01 mm precision. The mean data of a shrub during the growth season was examined in statistical analysis.

#### 2.3.5 Number of leaves

The number of leaves in each shrub was counted ever since the appearance of the first leaf up until the time of harvest. The mean data of a shrub during the growth season was examined in statistical analysis.

#### 2.3.6 Chlorophyll contents and the amount of carotenoid

To measure chlorophyll and carotene, first 0.1 g of completely developed young leaves were separated. Then, it was grinded in a porcelain mortar with 10 mL of 99% methanol in order to extract pigments followed by mixture in centrifuge for 5 minutes with a speed of 3000 rpm. The extent of absorption from the resulting extract were read at wavelengths including 470, 653, and 666 nm using a spectrophotometer (Bio Quest, CE 2502, UK). Finally, chlorophyll contents were calculated via the following relations (Dere et al., 1998):

CHLa = 15.65A666–7.340A653

CHLb = 27.05 A653–11.21 A666

CHLx + c = 1000 A470–2.860 CHLa - 129.2 CHLb

CHLt = CHLa + CHLb + CHLx + c

Total Carotenoid: CHLx + c, amount of chlorophyll b: CHLb, amount of chlorophyll a: CHLa, total chlorophyll: CHLt

#### 2.3.7 Total phenolic compounds

The Singleton and Rossi (1965) method was used to determine total phenolic compounds. Freeze-dried samples (50 mg) extracted with 10mL 80% methanol in 30°C water bath and shaken at 240 rpm overnight for 16–19 h. After filtering, 50μL of the methanolic extract was then mixed with 350μL of H_2_O and 200μL of 2N Folin- Ciocalteu reagents. The mixture was incubated for 1 h in 1mL of 10% Na_2_CO_3_ at 25 °C. Absorbance at 735 nm of the incubated mixture was then measured by a Beckman DU-64 spectrophotometer (Bio Quest, CE 2502, UK), with a standard curve to estimate Gallic acid equivalent concentrations.

#### 2.3.8 Anthocyanin contents

The Wagner (1979) method was used to measure the amount of anthocyanin contents in leaves. 0.1 g of the leave's texture was thoroughly grinded in a porcelain mortar with 10 mL of acidic methanol (pure methanol and hydrochloric acid with volume ratio of 1:99). The extract was then poured into screw-cap test tubes and was placed in darkness for 24 hours at 25°C. Next, it was centrifuged for 10 minutes at 4000 rpm. The absorption of the supernatant was measured at a wavelength of 550 nm. To calculate the concentration, the light extinction coefficient (ε) of 33000 M^−1^cm^−1^ was considered.

A = εbc

A = Absorption, b = cuvette width, c = the concentration of the intended solution

#### 2.3.9 Statistical analysis

There were 15 cress shrubs at each pot; the mean data of each pot during the growth period was examined in statistical analysis. The data were subjected to two-way analysis of variance (ANOVA) and the LSD test was used as a post-test. P ≥ 0.01 was considered not significant. Charts were drawn using Excel 2013 software.

### 2.4 Conclusion

#### 2.4.1 Effects of light treatments on plant growth and morphology

**Fig. 2:**
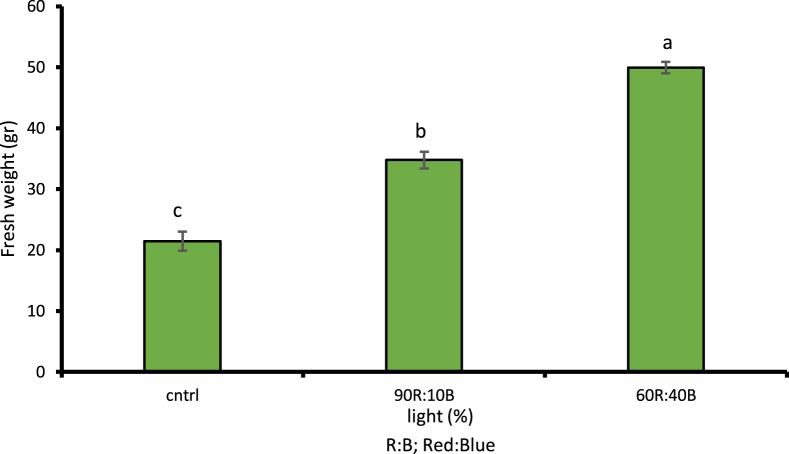
Comparison of the effect of optical spectra on fresh weight of leaf and stem (p ≤ 0.01). Different letters indicate significant differences between treatments by LSD test.

**Fig. 3:**
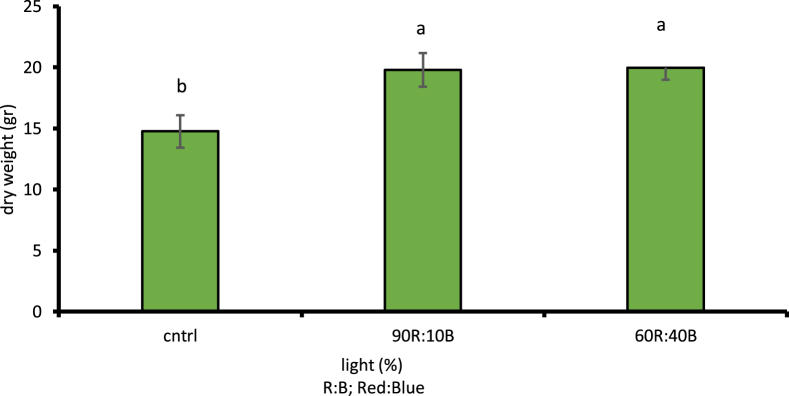
Comparison of the effect of optical spectra on dry weight of leaf and stem (p ≤ 0.01). Different letters indicate significant differences between treatments by LSD test.

**Fig. 4:**
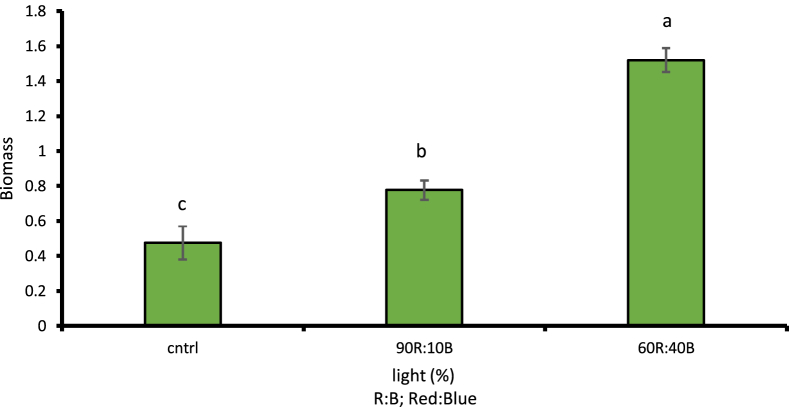
Comparison of the Effect of Optical Spectra on the Biomass of the Crop Plant (p ≤ 0.01). Different letters indicate significant differences between treatments by LSD test.

Assessment of different light treatments showed the effects of LED lights on the fresh and dry weights of cress as well as its biomass at 1% likelihood level; a considerable increase in the amount of fresh and dry weight of leaves, stems, and biomass of cress was observed compared to plants grown under natural sunlight conditions. According to LSD, 1% of the plant's fresh weight under 60R:40B treatment has 57.11% increase compared to natural light treatment (Fig. 2). Moreover, 26.06% increase was observed in the plant's dry weight under 90R:10B treatment compared to control sample (Fig. 3). The highest amount of biomass was observed under 60R:40B treatment as 1.51 g/kg of dry weight (Fig. 4).

**Fig. 5:**
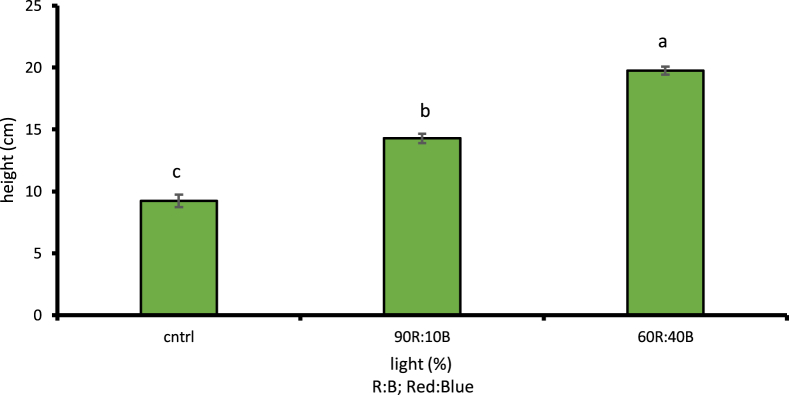
Comparison of the effect of optical spectra on plant height (p ≤ 0.01). Different letters indicate significant differences between treatments by LSD test.

**Fig. 6:**
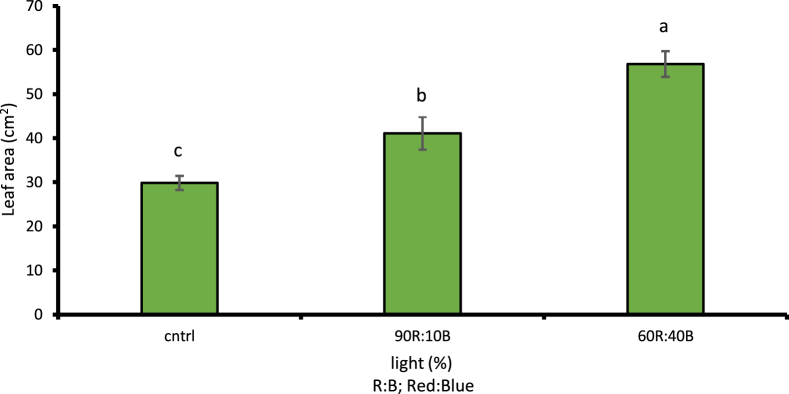
Comparison of the Effect of Optical Spectra on the Surface of the Leaf (p ≤ 0.01). Different letters indicate significant differences between treatments by LSD test.

**Fig. 7:**
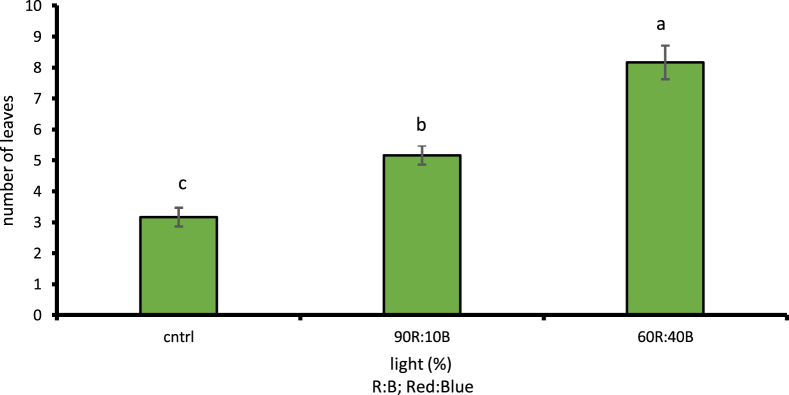
Comparison of the Effect of Optical Spectra on the number of leaves in crass (p ≤ 0.01). Different letters indicate significant differences between treatments by LSD test.

**Fig. 8:**
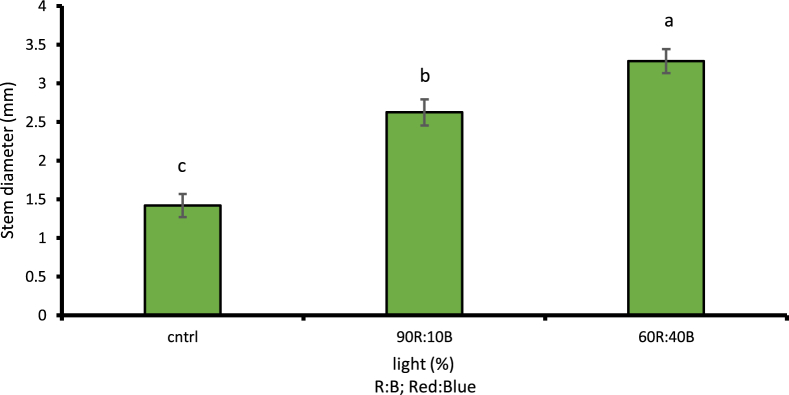
Comparison of the Effect of Optical Spectrometers on Stem diameter (p ≤ 0.01). Different letters indicate significant differences between treatments by LSD test.

Given the examinations done on different light treatments compared to control treatment, the cress plant had its maximum height under 60R:40B light at 19.76 cm with 1% level of likelihood which was a 53.28% increase compared to the control light treatment (Fig. 5). Under the 60R:40B light treatment, the cress leaf had the largest area at 56.78 cm2 with 1% level of likelihood, i.e. 47.46% increase compared to the control light treatment (Fig. 6). The stem diameter and the number of leaves at 1% level of likelihood under 60R:40B treatment had their maximum values as 3.28 mm and 8.16, respectively; both were in turn increased by 56.7 and 61.27% compared to control treatment (Figs. 7 and 8).

#### 2.4.2 Effects of light treatments on the biochemical parameter

##### 2.4.2.1 The amount of chlorophyll (a,b.T) and carotenoid

**Fig. 9:**
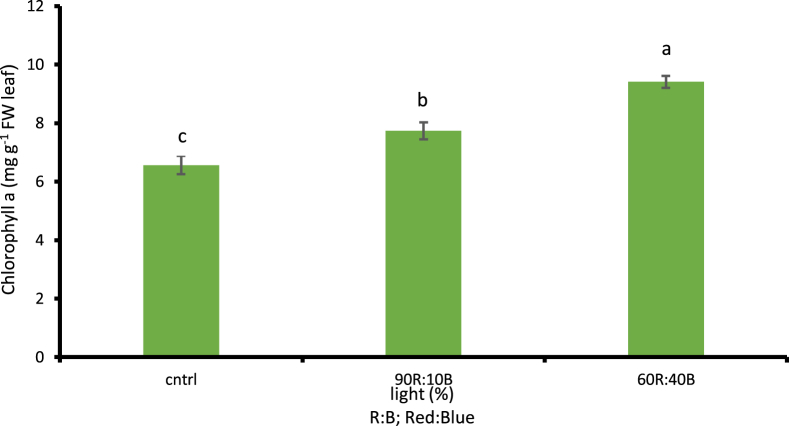
Comparison of the Effect of Optical Spectrometers on Chlorophyll a (p ≤ 0.01). Different letters indicate significant differences between treatments by LSD test.

**Fig. 10:**
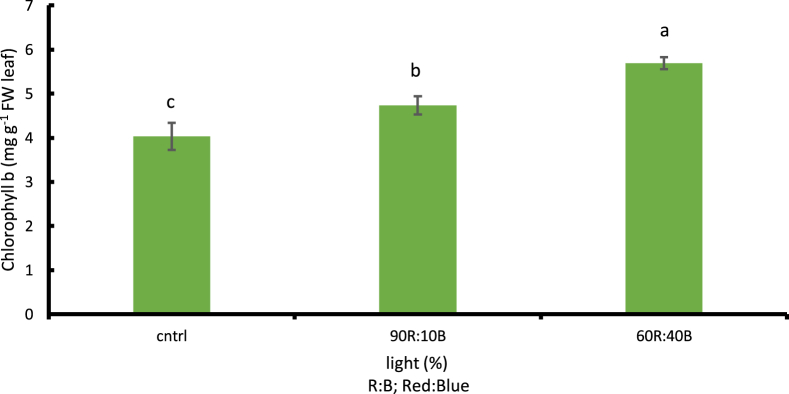
Comparison of the Effect of Optical Spectrometers on Chlorophyll b (p ≤ 0.01). Different letters indicate significant differences between treatments by LSD test.

**Fig. 11:**
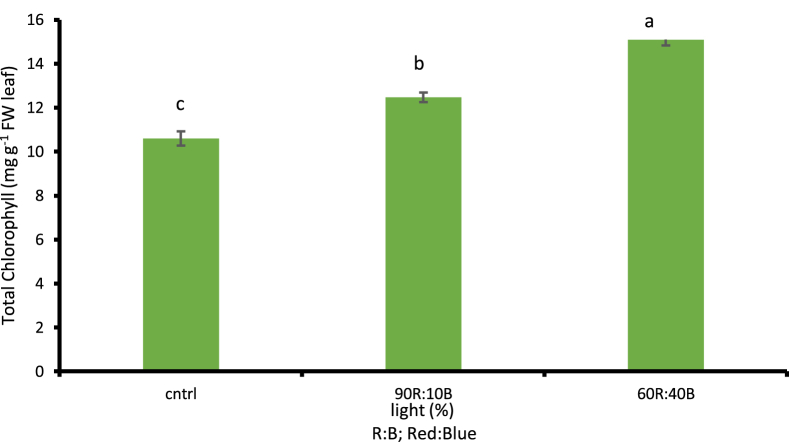
Comparison of the Effect of Optical Spectrometers on Total Chlorophyll (p ≤ 0.01). Different letters indicate significant differences between treatments by LSD test.

According to the obtained results, the more the percentage of the blue light, the more the amount of chlorophyll was considerably increased based on 1% LSD. The highest amount of chlorophyll a,b.T was observed under 60R:40B treatment with values of 9.4, 5.68, and 15.09 mg g^−1^ FW leaf, respectively (p ≤ 0.01). Given the comparison between light treatments and the control treatment with natural sunlight, the highest amounts were observed under lights with higher percentages of blue light while the lowest were observed under control treatment with values of 6.54 mg g^−1^ FW leaf, 4.03 mg g^−1^ FW leaf, and 10.59 mg g^−1^ FW leaf in chlorophyll a, b, and T, respectively. Compared to the control treatment, there was 30.42% increase in chlorophyll a (Fig. 9), 29.04% increase in chlorophyll b (Fig. 10) and 29.82% total chlorophyll (Fig. 11) based on 1% LSD. As for the amount of carotenoid, no significant difference was observed in the percentages of red and blue lights compared to control treatment.

##### 2.4.2.2 Total amount of phenol

**Fig. 12:**
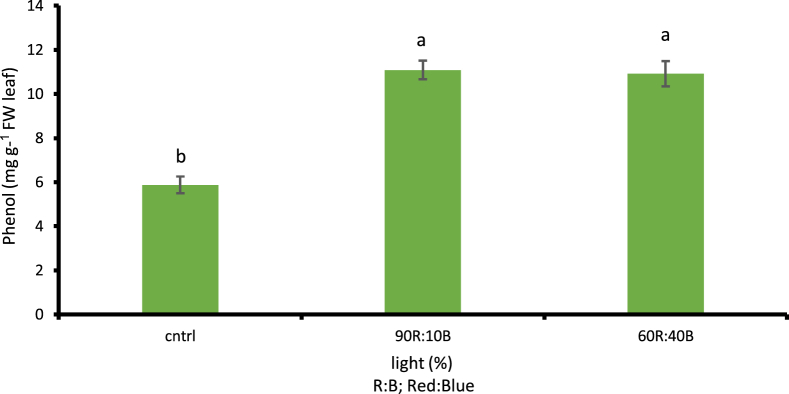
Comparison of the Effect of Optical Spectrometers on Phenol (p ≤ 0.01). Different letters indicate significant differences between treatments by LSD test.

Based on the results, the total amount of phenol under the effect of LED lamps during the growth period was significantly increased (p ≤ 0.01). Both 60R:40B and 90R:10B treatments were able to raise the amount of phenol up to 46.67% in cress, compared to the control treatment in which no artificial light was received and natural sunlight was used (Fig. 12).

##### 2.4.2.3 The amount of anthocyanin

**Fig. 13:**
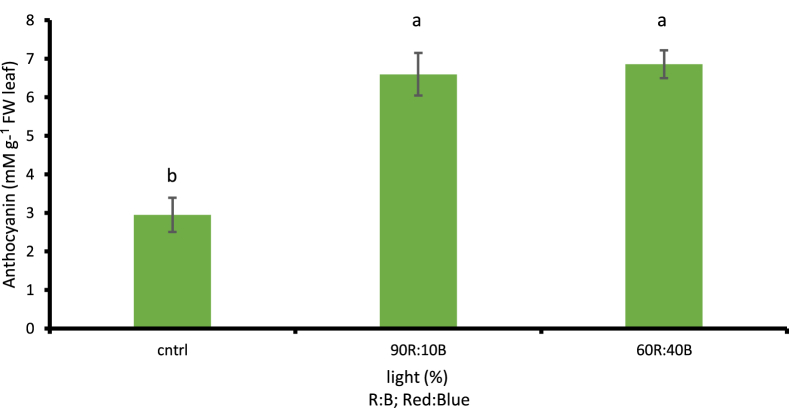
Comparison of the Effect of Optical Spectrometers on Anthocyanin (p ≤ 0.01). Different letters indicate significant differences between treatments by LSD test.

When LED lamps with different light percentages including 60R:40B and 90R:10B were used, considerable increase (p ≤ 0.01) was observed in the amount of anthocyanin in cress during growth period with respect to the control treatment (sunlight) (Fig. 13). This amount in cress was increased by 32.55% during the use of LED lamps compared to the control treatment. The minimum amount of anthocyanin under natural sunlight treatment was observed as 2.94 Mm g^−1^ FW leaf, with a significant difference compared to artificial light treatments (p ≤ 0.01).

### 2.5 Discussion

Light is considered as an important source for photosynthesis; however, photosynthesis can rely on a set of light regulators and sensors as well. Blue and red lights activate different light sensors and gene expressions that may positively or negatively affect the growth and development of plants (O'Carrigan et al., 2014). Consequently, it can be concluded that the presence of both wavelengths (blue and red) is necessary for plants; accordingly, the majority of research are now focused on achieving a suitable combination of these lights (Massa et al., 2008). Okamoto et al. (1996) reported that the presence of both the blue and red light is necessary and essential for photosynthesis as chlorophyll absorbs both of these wavelengths. They further suggested that the presence of blue light is beneficial for the morphology and the general health of plants. Overall, different wavelengths have been able to produce various effects on morphological and physiological characteristics as well as flowering capabilities and plant photosynthesis (Ménard et al., 2005). When LED lights are used, the ratio of blue light to red light is of substantial importance as the application of both wavelengths can increase the growth and function of plants by 20% compared to the use of each wavelength in isolation (Brazaitytė et al., 2009; GOTO, 2003; Lefsrud et al., 2008; Xiaoying et al., 2012; XiaoYing et al., 2011).

#### 2.5.1 Growth and morphological characteristics

Table 1 demonstrates the measured traits of morphological and growing characteristics of cress. According to the results of the present study, the application of LED lamps in blue and red spectra has a significant effect on the morphological traits of cress. Among the treatments applied, the 60R:40B treatment had the highest effect on traits including fresh weight, biomass, plant's height, area of leaf, number of leaves, and stem diameter of cress, with significant differences compared to treatments without blue and red spectra (p ≤ 0.01). Dry weights of cress did not have a significant difference under various treatments, though a considerable, significant difference was observed compared to the control treatment (p ≤ 0.01). Phytochromes along with cryptochromes result in photomorphogenesis in plants; therefore, the study of light waves becomes substantially important. Moreover, more suitable growth responses can be obtained by taking the maximum light absorption by receptors into account when choosing the spectral quality of light (Pinho et al., 2012).

**Table 1:** Average values (n = 10 ± SE) followed by different letters within a row differ significantly (a = 0.01) according to LSD criterion.

Parameters	Light treatments		
	90R:10B	60R:40B	Control
Fresh weight (gr)	34.78 ± 1.36 b	49.95 ± 0.96a	21.42 ± 1.57 c
Dry weight (gr)	19.78 ± 1.36 a	19.95 ± 0.96 a	14.75 ± 1.33 b
Biomass	0.77 ± 0.05 b	1.5 ± 0.06 a	0.47 ± 0.09 c
Height (cm)	14.28 ± 0.36 b	19.76 ± 0.32 a	9.23 ± 0.49 c
Leaf area (cm^2^)	41.09 ± 3.72 b	56.78 ± 2.91 a	29.83 ± 1.57 c
Leaf Number	5.1 ± 0.43 b	8.16 ± 0.54 a	3.16 ± 0.37 c
Stem diameter (mm)	2.62 ± 0.16 b	3.28 ± 0.15 a	1.42 ± 0.15 c

* Numbers with dissimilar letters in each row have significant differences at the1% probability level using LSD test.

Many growth parameters such as the plant's fresh and dry weights, stem length and area of leaves are affected through impacts on phytochrome receptors along with red light spectrum (Whitelam and Halliday, 2007). For instance, the application of red light increase the area of leaves in cucumber plants (Hogewoning et al., 2010). Moreover, in a plant such as Scots pine, increase in the extent of applied red light increased its biomass (Riikonen et al., 2016); similar results were observed in the study conducted on cress. Albeit, in other studies, the addition of blue light to red was apparently essential for photosynthesis system activity and enables the production of more biomass in plants (Hogewoning et al., 2010; Johkan et al., 2010). Accordingly, in this experiment, various percentages of blue light were applied alongside red light so that the plant does not suffer from deficiency in terms of growth conditions. As the results showed, the use of a combination of blue and red lights produced the best result in morphological characteristics of cress compared to the control treatment. The application of red and blue lights improved the plant's growth in terms of the fresh weight of the stem and leaves compared to natural sunlight which were also consistent with the results of a study by Randall and Lopez (2014). The highest fresh weight caused by the application of red and blue light was reported in daisies (Kim et al., 2004). The highest fresh weight in lettuce was also reported under blue and red light treatment (Johkan et al., 2010). The use of red light alongside blue light yielded better results in increasing the dry weight of needle leaves in Norwegian and Scots pine seedlings (Riikonen et al., 2016). Moreover, the application of red and blue lights led to increase in the dry weight of leaves and stems of common sage, lettuce, radish, pepper, and spinach (Heo et al., 2002; Yorio et al., 2001; Brown et al., 1995; Pinho et al., 2004). At the chloroplast level, blue light has a high photosynthetic capacity with the expression of features similar to sunlight (XiaoYing et al., 2011). Subsequently, it can be concluded that photosynthesis is increased when affected by blue light which may be due to the specific sensitivity of cryptochromes and phototropins to blue light (Whitelam and Halliday, 2007); such an increase in photosynthesis, in turn, results in more vegetative growth in plants. With increased growth, increase in the stem's diameter and height as well as the area and number of leaves can be expected. The effect of 60R:40B treatment on increasing morphological characteristics of cress among other light treatments is easily witnessed. The effect of blue and red light on changing the height of petunia plant is caused by the effect of blue light and excitation of cryptochromes which would lead to the production of signals that stimulate gibberellin production and as a result, increasing the stem length; this alters the extent of blue light's presence in the environment and its increase or decrease results in changes in gibberellin excretion and subsequently, changes in height (Fukuda et al., 2016). The use of blue light alone increased the height of basil medicinal plant (Glowacka, 2008). As Fukuda et al. (2016) suggested, cytokinins are activated as the extent of photosynthesis raises in leaves under blue light treatment. Such an increase in height enhances the performance of products at the time of harvest; meanwhile, short heights in plants reduce the quality of the product in terms of market interest and places the harvest procedure at risk (Kong et al., 2019). Therefore, adjusting the lighting quality at the time of planting could introduce numerous morphological changes in plants (Demotes Mainard et al., 2016; Mah et al., 2018). Similar to the results of this study with respect to increase in the area of leaves, same results were reported in leaves of lettuce, radish, soy, wheat and roses with the application of both the blue and red lights (Stutte et al., 2009; Samuolienė et al., 2011; Cope and Bugbee, 2013; Terfa et al., 2013). Given the examinations conducted on lettuce, those plants that did not receive any blue light had a smaller area of leaves compared to plants that received even small extents of blue light, with 66% increase in the area of leaves (Clavijo-Herrera et al., 2018). In general, if exposure to light results in biochemical and physiological changes in plants, then these changes can be associated with changes in morphological and anatomic structure of leaves, particularly the anatomic components of leave (Puglielli et al., 2017). Fig. 8 shows the increase in stem's diameter during the growth period caused by exposure to LED lights where the percentage of blue light was higher than other treatments. Consistent with the results of this study, Glowacka (2004) reported increase in stem's diameter under blue light coverage. Researchers believe that blue light is involved in a vast spectrum of vegetative process such as photosynthesis performance of leaves and morphological structures of plants; this could also lead to an increase in the number of leaves in a vast number of plants (Brown et al., 1995; Bukhov et al., 1995; Hogewoning et al., 2010; Matsuda et al., 2004, 2008; Ohashi-Kaneko et al., 2006; Whitelam and Halliday, 2007; Yorio et al., 1998).

#### 2.5.2 Biochemical characteristics

Results listed in Table 2 show the significant effect of blue and red light on biochemical characteristics measure in the cress plant. According to statistical results, the application of both blue and red light can increase the biochemical characteristics in cress compared to natural sunlight. Epidemiologic and experimental studies have shown that increased consumption of fruits and vegetables enhances human health and prevents cancer due to high amounts of fibers, vitamins, minerals and phytochemicals (Slavin and Lloyd, 2012). Vitamins and green pigments (high chlorophyll) are valuable compounds in this plant which play an important role in human health (Curran-Celentano et al., 2001; Giovannucci, 1999; Humphrey et al., 1992; Mayne, 1996; Seddon et al., 1994). Light adjustment can be pointed out as one of the strategic tools during cultivation for managing such valuable components in fruits and vegetables (Riga et al., 2014). Consequently, it can be predicted that the quality of light has an effective role in chlorophyll synthesis and accumulation of materials including phenols and anthocyanin. In this experiment, the highest amounts of chlorophyll, phenol, and anthocyanin were observed in treatments where blue and red light were applied during growth. Photosynthetic photon flux density (PPFD) and daily period duration are two major components in the adjustment of plant growth and development and nutritional value (Kang et al., 2013; Zhang et al., 2018). For instance, the quality of light balanced photochemical characteristics of lettuce (Li and Kubota, 2009). Quality and intensity of light are also two effective factors on photosynthetic pigments (Hogewoning et al., 2010; Gutu et al., 2013); among monochrome spectra, the constant application of blue and red spectra have a positive effect on the growth of chloroplast and leaves in mesophyll (Liu et al., 2011). In this regard, studies conducted on plants such as lettuce and cucumber showed that the use of a suitable combination of blue and red lights would accelerate growth and photosynthesis (Hogewoning et al., 2010; Xu et al., 2015). As with research on cucumber, the application of blue and red lights increased the amounts of chlorophyll a, b, and T compared to control treatment which was also consistent with the results of this study (Hogewoning et al., 2010; Hernández and Kubota, 2016). In general, a proper combination of red and blue lights can increase the amounts of chlorophyll a an b which can be a suitable approach to confront tensions and damages caused by free radicals (Johkan et al., 2010; Schagerl and Müller, 2006). According to available reports, blue light enables chlorophyll photosynthesis and opens pores (Urbonavičiūtė et al., 2007). Furthermore, blue light increases the amount of chlorophyll and photosynthesis by 30%, based on the type of the plant (Frechijia et al., 1999). To this end, an examination by Yanagi et al. (1996) demonstrated the positive effect of blue light in cryptochromes system activity which increases the amount of chlorophyll. Wu et al. (2007) also showed that the blue light spectrum increases the amount of chlorophyll in green pea plant. The results of other studies are also in line with those obtained in the present research. The measurement results of phenol contents and the amount of anthocyanin (Table 2) showed that cress pots under red and blue light treatment produced higher amounts of phenol and anthocyanin compared to control treatment. Anthocyanin is a group of plant pigments which are capable of production and accumulation in response to light stimulants (Liu et al., 2018a,b). The majority of studies on the effects of light quality on phenolic compounds, especially anthocyanin and other flavonoids were conducted prior to 2013 (Chen and Murata, 2011). Considering the observations carried out on plants such as parsley, basil, and tomato leaves, it was stated that the application of blue light during growth could bring about considerable increase in the amounts of phenolic compounds (Giri, 2011).

**Table 2:** Average values (n = 10 ± SE) followed by different letters within a row differ significantly (a = 0.01) according to LSD criterion.

Parameters	Light treatments		
	90R:10B	60R:40B	Control
Chlorophyll a (mg ^g−1^ FW leaf)	7.73 ± 0.29 b	9.4 ± 0.2 a	6.56 ± 0.3 c
Chlorophyll b (mg ^g−1^ FW leaf)	4.73 ± 0.2 b	5.68 ± 0.13 a	4.03 ± 0.31 c
Total chlorophyll (mg ^g−1^ FW leaf)	12.47 ± 0.21 b	15.09 ± 0.26 a	10.59 ± 0.32 c
Total phenol (mg ^g−1^ FW leaf)	11.08 ± 0.42 a	10.91 ± 0.57 a	5.78 ± 0.38 b
Anthocyanin (mM g-^1^ FW leaf)	6.59 ± 0.55 a	6.85 ± 0.39 a	2.94 ± 0.44 b

* Numbers with dissimilar letters in each row have significantdifferences at the1% probability level using LSD test.

Light has a direct impact on growth, distinction, and synthesis of phytochemicals in the majority of plants (Fazal et al., 2016). Consequently, changes in the density and quality of light results in a set of changes in certain biochemical and physiological process in plants which are observable through its reflection in morphological and anatomic parameters. Light is the main source for the absorption of photosynthetic carbon in plants which, accordingly, is an important factor in regulation and photosynthesis of phytochemicals (Khan et al., 2013). There are various studies that show the fact that different wavelengths of light result in an increase in the biosynthesis of different phytochemicals in plants (Taulavuori et al., 2017; Arena et al., 2016; Craver et al., 2017).

In general, the red light spectrum (625–700 nm) increases phenolic compounds in green vegetables (Olle and Viršile, 2013). There are numerous studies suggesting the increase in total amounts of phenolic compounds as well as anthocyanin; overall, the use of LED lamps at the planting period can activate secondary metabolic paths in plants (Routray et al., 2018). In greenhouse cultivation of lettuce, it was observed that certain wavelengths (blue and red) are of considerable superiority over other wavelengths in activating phenol synthetic paths and its storage (Samuolienė et al., 2012). In the red-leaf lettuce, the anthocyanin contents were considerably affected by red light (Johken et al., 2010). Moreover, it was shown that the red light spectrum has a substantial effect on the synthesis of anthocyanin along with regulating phytochromes (Ninu et al., 1999; Giliberto et al., 2005). Accordingly, with respect to the effective impact of red light in anthocyanin synthesis in plants, an American company has mentioned the red light as the best stimulant for anthocyanin production compared to other light spectrums (Zhou and Singh, 2002). The results obtained in this study are similar to those of Bantis et al. (2018).

### 2.6 Overall conclusion

In this study, the entire morphological traits examined in cress were placed under red and blue spectrum LED lamps; each wavelengths had its own particular effect in their corresponding receptors in the plant, ultimately increasing the extent of growth and performance of the plant. As expected, the combination of blue and red lights as effective wavelengths on plant's growth had considerable effects on the vegetative traits compared to the control treatment (sunlight). Therefore, it can be expressed that the presence of both wavelengths (blue and red) is necessary for a better and more complete growth of the plant; subsequently, proper percentages of the combination of these two wavelengths should be found. The results of this study showed that the biochemical characteristics of cress under LED light coverage (red and blue combination) was superior over natural conditions, which equally increases this plant's properties in terms of human health. Finally, it can be suggested that the use of these lamps can be possible in line with better economic production within controlled conditions (greenhouse).

## Declarations

### Author contribution statement

L. Ajdanian: Performed the experiments; Analyzed and interpreted the data; Contributed reagents, materials, analysis tools or data; Wrote the paper.

M. Babaei: Analyzed and interpreted the data; Contributed reagents, materials, analysis tools or data.

H. Arouei: Conceived and designed the experiments; Contributed reagents, materials, analysis tools or data.

### Funding statement

This research did not receive any specific grant from funding agencies in the public, commercial, or not-for-profit sectors.

## Competing interest statement

The authors declare no conflict of interest.

### Additional information

No additional information is available for this paper.

## References

[bib1] Arena C., Tsonev T., Doneva D., De Micco V., Michelozzi M., Brunetti C., Loreto F. (2016). The effect of light quality on growth, photosynthesis, leaf anatomy and volatile isoprenoids of a monoterpene-emitting herbaceous species (Solanum lycopersicum L.) and an isoprene-emitting tree (Platanus orientalis L.). Environ. Exp. Bot..

[bib2] Bantis F., Karamanoli K., Ainalidou A., Radoglou K., Constantinidou H.-I.A. (2018). Light emitting diodes (LEDs) affect morphological, physiological and phytochemical characteristics of pomegranate seedlings. Sci. Hortic..

[bib3] Brazaitytė A., Duchovskis P., Urbonavičiūtė A., Samuolienė G., Jankauskienė J., Kazėnas V., Breivė K. (2009). After-effect of light-emitting diodes lighting on tomato growth and yield in greenhouse. Sodininkystė ir daržininkystė.

[bib4] Briggs W.R., Huala E. (1999). Blue-light photoreceptors in higher plants. Annu. Rev. Cell Dev. Biol..

[bib5] Brown C.S., Schuerger A.C., Sager J.C. (1995). Growth and photomorphogenesis of pepper plants under red light-emitting diodes with supplemental blue or far-red lighting. J. Am. Soc. Hortic. Sci..

[bib6] Bukhov N., Drozdova I., Bondar V. (1995). Light response curves of photosynthesis in leaves of sun-type and shade-type plants grown in blue or red light. J. Photochem. Photobiol. B Biol..

[bib7] Bula R., Morrow R., Tibbitts T., Barta D., Ignatius R., Martin T. (1991). Light-emitting diodes as a radiation source for plants. Hortscience.

[bib8] Cashmore A.R., Jarillo J.A., Wu Y.-J., Liu D. (1999). Cryptochromes: blue light receptors for plants and animals. Science.

[bib9] Chen T.H., Murata N. (2011). Glycinebetaine protects plants against abiotic stress: mechanisms and biotechnological applications. Plant Cell Environ..

[bib10] Choi H.G., Moon B.Y., Kang N.J. (2015). Effects of LED light on the production of strawberry during cultivation in a plastic greenhouse and in a growth chamber. Sci. Hortic..

[bib11] Clavijo-Herrera J., van Santen E., Gómez C. (2018). Growth, water-use efficiency, stomatal conductance, and nitrogen uptake of two lettuce cultivars grown under different percentages of blue and red light. Horticulturae.

[bib12] Cope K.R., Bugbee B. (2013). Spectral effects of three types of white light-emitting diodes on plant growth and development: absolute versus relative amounts of blue light. Hortscience.

[bib13] Craver J.K., Gerovac J.R., Lopez R.G., Kopsell D.A. (2017). Light intensity and light quality from sole-source light-emitting diodes impact phytochemical concentrations within Brassica microgreens. J. Am. Soc. Hortic. Sci..

[bib14] Curran-Celentano J., Hammond B.R., Ciulla T.A., Cooper D.A., Pratt L.M., Danis R.B. (2001). Relation between dietary intake, serum concentrations, and retinal concentrations of lutein and zeaxanthin in adults in a Midwest population. Am. J. Clin. Nutr..

[bib15] Currey C.J., Lopez R.G. (2013). Cuttings of Impatiens, Pelargonium, and Petunia propagated under light-emitting diodes and high-pressure sodium lamps have comparable growth, morphology, gas exchange, and post-transplant performance. Hortscience.

[bib16] Demotes-Mainard S., Péron T., Corot A., Bertheloot J., Le Gourrierec J., Pelleschi-Travier S., Boumaza R. (2016). Plant responses to red and far-red lights, applications in horticulture. Environ. Exp. Bot..

[bib96] Dere Ş., Güneş T., Sivaci R. (1998). Spectrophotometric determination of chlorophyll-A, B and total carotenoid contents of some algae species using different solvents. Turk. J. Bot..

[bib17] Fazal H., Abbasi B.H., Ahmad N., Ali M., Ali S. (2016). Sucrose induced osmotic stress and photoperiod regimes enhanced the biomass and production of antioxidant secondary metabolites in shake-flask suspension cultures of Prunella vulgaris L. Plant Cell Tissue Organ Cult..

[bib18] Folta K.M., Childers K.S. (2008). Light as a growth regulator: controlling plant biology with narrow-bandwidth solid-state lighting systems. Hortscience.

[bib19] Franklin K.A., Whitelam G.C. (2005). Phytochromes and shade-avoidance responses in plants. Ann. Bot..

[bib20] Frąszczak B., Golcz A., Zawirska-Wojtasiak R., Janowska B. (2014). Growth rate of sweet basil and lemon balm plants grown under fluorescent lamps and LED modules. Acta Sci. Pol. Hortorum Cultus.

[bib21] Frechijia S., Zhu J., Talbott L.D., Zeiger E. (1999). Stomata from npq1, a zeaxanthin-less Arabidopsis mutant, lack a specific response to blue light. Plant Cell Physiol..

[bib22] Fukuda N., Ajima C., Yukawa T., Olsen J.E. (2016). Antagonistic action of blue and red light on shoot elongation in petunia depends on gibberellin, but the effects on flowering are not generally linked to gibberellin. Environ. Exp. Bot..

[bib23] Gautam P., Terfa M.T., Olsen J.E., Torre S. (2015). Red and blue light effects on morphology and flowering of Petunia× hybrida. Sci. Hortic..

[bib24] Giliberto L., Perrotta G., Pallara P., Weller J.L., Fraser P.D., Bramley P.M., . . . Giuliano G. (2005). Manipulation of the blue light photoreceptor cryptochrome 2 in tomato affects vegetative development, flowering time, and fruit antioxidant content. Plant Physiol..

[bib25] Giovannucci E. (1999). Tomatoes, tomato-based products, lycopene, and cancer: review of the epidemiologic literature. J. Natl. Cancer Inst..

[bib26] Giri J. (2011). Glycinebetaine and abiotic stress tolerance in plants. Plant Signal. Behav..

[bib27] Glowacka B. (2008). Wpływ składu spektralnego światła na wzrost rozsady bazylii pospolitej (Ocimum basilicum L.), melisy lekarskiej (Melissa officinalis L.) i ogórecznika lekarskiego (Borago officinalis L.). Zesz. Probl. Postepow Nauk. Rol..

[bib29] Gómez C., Mitchell C. (2016). Search of an Optimized Supplemental Lighting Spectrum for Greenhouse Tomato Production with Intracanopy Lighting. Paper Presented at the VIII International Symposium on Light in Horticulture 1134.

[bib30] GOTO E. (2003). Effects of light quality on growth of crop plants under artificial lighting. Environ. Control Biol..

[bib31] Gutu A., Nesbit A.D., Alverson A.J., Palmer J.D., Kehoe D.M. (2013). Unique role for translation initiation factor 3 in the light color regulation of photosynthetic gene expression. Proc. Natl. Acad. Sci..

[bib32] Hao X., Little C., Khosla S. (2012). LED inter-lighting in year-round greenhouse mini-cucumber production. Paper Presented at the VII International Symposium on Light in Horticultural Systems 956.

[bib33] Heo J., Lee C., Chakrabarty D., Paek K. (2002). Growth responses of marigold and salvia bedding plants as affected by monochromic or mixture radiation provided by a light-emitting diode (LED). Plant Growth Regul..

[bib34] Hernández R., Kubota C. (2012). Tomato seedling growth and morphological responses to supplemental LED lighting red: blue ratios under varied daily solar light integrals. Paper Presented at the VII International Symposium on Light in Horticultural Systems 956.

[bib35] Hernández R., Kubota C. (2016). Physiological responses of cucumber seedlings under different blue and red photon flux ratios using LEDs. Environ. Exp. Bot..

[bib36] Hirai T., Amaki W., Watanabe H. (2006). Action of blue or red monochromatic light on stem internodal growth depends on plant species. Paper Presented at the V International Symposium on Artificial Lighting in Horticulture 711.

[bib37] Hogewoning S.W., Trouwborst G., Maljaars H., Poorter H., van Ieperen W., Harbinson J. (2010). Blue light dose–responses of leaf photosynthesis, morphology, and chemical composition of Cucumis sativus grown under different combinations of red and blue light. J. Exp. Bot..

[bib38] Huché-Thélier L., Crespel L., Le Gourrierec J., Morel P., Sakr S., Leduc N. (2016). Light signaling and plant responses to blue and UV radiations—perspectives for applications in horticulture. Environ. Exp. Bot..

[bib39] Humphrey J., West K., Sommer A. (1992). Vitamin A deficiency and attributable mortality among under-5-year-olds. Bull. World Health Organ..

[bib40] Jeong S.W., Hogewoning S.W., van Ieperen W. (2014). Responses of supplemental blue light on flowering and stem extension growth of cut chrysanthemum. Sci. Hortic..

[bib41] Johkan M., Shoji K., Goto F., Hashida S.-n., Yoshihara T. (2010). Blue light-emitting diode light irradiation of seedlings improves seedling quality and growth after transplanting in red leaf lettuce. Hortscience.

[bib42] Joshi N.C., Ratner K., Eidelman O., Bednarczyk D., Zur N., Many Y., Gilad Z. (2019). Effects of daytime intra-canopy LED illumination on photosynthesis and productivity of bell pepper grown in protected cultivation. Sci. Hortic..

[bib43] Kang J.H., KrishnaKumar S., Atulba S.L.S., Jeong B.R., Hwang S.J. (2013). Light intensity and photoperiod influence the growth and development of hydroponically grown leaf lettuce in a closed-type plant factory system. Hortic, Environ., Biotechnol..

[bib44] Khan M.A., Abbasi B.H., Ahmed N., Ali H. (2013). Effects of light regimes on in vitro seed germination and silymarin content in Silybum marianum. Ind. Crops Prod..

[bib45] Kim H.-H., Goins G.D., Wheeler R.M., Sager J.C. (2004). Green-light supplementation for enhanced lettuce growth under red-and blue-light-emitting diodes. Hortscience.

[bib46] Kong Y., Schiestel K., Zheng Y. (2019). Pure blue light effects on growth and morphology are slightly changed by adding low-level UVA or far-red light: a comparison with red light in four microgreen species. Environ. Exp. Bot..

[bib47] Lefsrud M.G., Kopsell D.A., Sams C.E. (2008). Irradiance from distinct wavelength light-emitting diodes affect secondary metabolites in kale. Hortscience.

[bib48] Li Q., Kubota C. (2009). Effects of supplemental light quality on growth and phytochemicals of baby leaf lettuce. Environ. Exp. Bot..

[bib97] Liu X.Y., Guo S.R., Xu Z.G., Jiao X.L., Tezuka T. (2011). Regulation of chloroplast ultrastructure, cross-section anatomy of leaves, and morphology of stomata of cherry tomato by different light irradiations of light-emitting diodes. Hortscience.

[bib49] Liu X., Jiao X., Chang T., Guo S., Xu Z. (2018). Photosynthesis and leaf development of cherry tomato seedlings under different LED-based blue and red photon flux ratios. Photosynthetica.

[bib50] Liu Y., Tikunov Y., Schouten R.E., Marcelis L.F., Visser R.G., Bovy A. (2018). Anthocyanin biosynthesis and degradation mechanisms in Solanaceous vegetables: a review. Front. Chem..

[bib51] Lu N., MARUO T., JOHKAN M., HOHJO M., TSUKAGOSHI S., ITO Y., SHINOHARA Y. (2012). Effects of supplemental lighting with light-emitting diodes (LEDs) on tomato yield and quality of single-truss tomato plants grown at high planting density. Environ. Control Biol..

[bib52] Mah J.J., Llewellyn D., Zheng Y. (2018). Morphology and flowering responses of four bedding plant species to a range of red to far red ratios. Hortscience.

[bib53] Massa G.D., Kim H.-H., Wheeler R.M., Mitchell C.A. (2008). Plant productivity in response to LED lighting. Hortscience.

[bib98] Matsuda R., Ohashi-Kaneko K., Fujiwara K., Goto E., Kurata K. (2004). Photosynthetic characteristics of rice leaves grown under red light with or without supplemental blue light. Plant Cell Physiol..

[bib54] Matsuda R., Ohashi-Kaneko K., Fujiwara K., Kurata K. (2008). Effects of blue light deficiency on acclimation of light energy partitioning in PSII and CO2 assimilation capacity to high irradiance in spinach leaves. Plant Cell Physiol..

[bib55] Mayne S.T. (1996). Beta-carotene, carotenoids, and disease prevention in humans. FASEB J..

[bib56] Ménard C., Dorais M., Hovi T., Gosselin A. (2005). Developmental and physiological responses of tomato and cucumber to additional blue light. Paper Presented at the V International Symposium on Artificial Lighting in Horticulture 711.

[bib57] Morrow R.C. (2008). LED lighting in horticulture. Hortscience.

[bib58] Ninu L., Ahmad M., Miarelli C., Cashmore A.R., Giuliano G. (1999). Cryptochrome 1 controls tomato development in response to blue light. Plant J..

[bib59] O'Carrigan A., Babla M., Wang F., Liu X., Mak M., Thomas R. (2014). Analysis of gas exchange, stomatal behaviour and micronutrients uncovers dynamic response and adaptation of tomato plants to monochromatic light treatments. Plant Physiol. Biochem..

[bib60] Ohashi-Kaneko K., Matsuda R., Goto E., Fujiwara K., Kurata K. (2006). Growth of rice plants under red light with or without supplemental blue light. Soil Sci. Plant Nutr..

[bib61] Okamoto K., Yanagi T., Takita S., Tanaka M., Higuchi T., Ushida Y., Watanabe H. (1996). Development of plant growth apparatus using blue and red LED as artificial light source.

[bib62] Olle M., Viršile A. (2013). The effects of light-emitting diode lighting on greenhouse plant growth and quality. Agric. Food Sci..

[bib63] Pinho P. (2008). Usage and Control of Solid-State Lighting for Plant Growth.

[bib64] Pinho P., Jokinen K., Halonen L. (2012). Horticultural lighting–present and future challenges. Light. Res. Technol..

[bib65] Pinho P., Moisio O., Tetri E., Halonen L. (2004). Photobiological aspects of crop plants grown under light emitting diodes. Paper Presented at the Proceedings of the CIE Symposium.

[bib66] Pinho P., Tahvonen R., Lukkala R., Särkkä L., Halonen L., Tetri E. (2007). Evaluation of Lettuce Growth under Multi-Spectral-Component Supplemental Solid State Lighting in Greenhouse Environment.

[bib67] Puglielli G., Varone L., Gratani L., Catoni R. (2017). Specific leaf area variations drive acclimation of Cistus salvifolius in different light environments. Photosynthetica.

[bib68] Randall W.C., Lopez R.G. (2014). Comparison of supplemental lighting from high-pressure sodium lamps and light-emitting diodes during bedding plant seedling production. Hortscience.

[bib69] Riga P., Medina S., García-Flores L.A., Gil-Izquierdo Á. (2014). Melatonin content of pepper and tomato fruits: effects of cultivar and solar radiation. Food Chem..

[bib70] Riikonen J., Kettunen N., Gritsevich M., Hakala T., Särkkä L., Tahvonen R. (2016). Growth and development of Norway spruce and Scots pine seedlings under different light spectra. Environ. Exp. Bot..

[bib71] Routray W., Orsat V., Lefsrud M. (2018). Effect of postharvest LED application on phenolic and antioxidant components of blueberry leaves. ChemEng..

[bib72] Samuolienė G., Sirtautas R., Brazaitytė A., Duchovskis P. (2012). LED lighting and seasonality effects antioxidant properties of baby leaf lettuce. Food Chem..

[bib73] Samuolienė G., Sirtautas R., Brazaitytė A., Sakalauskaitė J., Sakalauskienė S., Duchovskis P. (2011). The impact of red and blue light-emitting diode illumination on radish physiological indices. Open Life Sci..

[bib74] Schagerl M., Müller B. (2006). Acclimation of chlorophyll a and carotenoid levels to different irradiances in four freshwater cyanobacteria. J. Plant Physiol..

[bib75] Seddon J.M., Ajani U.A., Sperduto R.D., Hiller R., Blair N., Burton T.C., Miller D.T. (1994). Dietary carotenoids, vitamins A, C, and E, and advanced age-related macular degeneration. JAMA.

[bib76] Singleton V.L., Rossi J.A. (1965). Colorimetry of total phenolics with phosphomolybdic-phosphotungstic acid reagents. Am. J. Enol. Vitic..

[bib77] Slavin J.L., Lloyd B. (2012). Health benefits of fruits and vegetables. Adv. Nutr..

[bib78] Stutte G.W., Edney S., Skerritt T. (2009). Photoregulation of bioprotectant content of red leaf lettuce with light-emitting diodes. Hortscience.

[bib80] Taulavuori E., Taulavuori K., Holopainen J.K., Julkunen-Tiitto R., Acar C., Dincer I. (2017). Targeted use of LEDs in improvement of production efficiency through phytochemical enrichment. J. Sci. Food Agric..

[bib81] Terfa M.T., Solhaug K.A., Gislerød H.R., Olsen J.E., Torre S. (2013). A high proportion of blue light increases the photosynthesis capacity and leaf formation rate of Rosa× hybrida but does not affect time to flower opening. Physiol. Plant..

[bib82] Urbonavičiūtė A., Pinho P., Samuolienė G., Duchovskis P., Vitta P., Stonkus A., Halonen L. (2007). Effect of short-wavelength light on lettuce growth and nutritional quality. Sodininkystė ir daržininkystė.

[bib83] Vänninen I., Pinto D., Nissinen A., Johansen N., Shipp L. (2010). In the light of new greenhouse technologies: 1. Plant-mediated effects of artificial lighting on arthropods and tritrophic interactions. Ann. Appl. Biol..

[bib84] Wagner G.J. (1979). Content and vacuole/extravacuole distribution of neutral sugars, free amino acids, and anthocyanin in protoplasts. Plant Physiol..

[bib85] Whitelam G.C., Halliday K.J. (2007). Light and Plant Development.

[bib86] Wu M.-C., Hou C.-Y., Jiang C.-M., Wang Y.-T., Wang C.-Y., Chen H.-H., Chang H.-M. (2007). A novel approach of LED light radiation improves the antioxidant activity of pea seedlings. Food Chem..

[bib87] Xiaoying L., Shirong G., Taotao C., Zhigang X., Tezuka T. (2012). Regulation of the growth and photosynthesis of cherry tomato seedlings by different light irradiations of light emitting diodes (LED). Afr. J. Biotechnol..

[bib88] XiaoYing L., ShiRong G., ZhiGang X., XueLei J., Tezuka T. (2011). Regulation of chloroplast ultrastructure, cross-section anatomy of leaves, and morphology of stomata of cherry tomato by different light irradiations of light-emitting diodes. Hortscience.

[bib89] Xu W., Liu X., Jiao X., Xu Z. (2015). Effect of blue light quantity on growth and quality of lettuce. J. Nanjing Agric. Univ..

[bib90] Yanagi T., Okamoto K., Takita S. (1996). Effects of blue, red, and blue/red lights of two different PPF levels on growth and morphogenesis of lettuce plants.

[bib91] Yeh N., Chung J.-P. (2009). High-brightness LEDs—energy efficient lighting sources and their potential in indoor plant cultivation. Renew. Sustain. Energy Rev..

[bib92] Yorio N.C., Goins G.D., Kagie H.R., Wheeler R.M., Sager J.C. (2001). Improving spinach, radish, and lettuce growth under red light-emitting diodes (LEDs) with blue light supplementation. Hortscience.

[bib93] Yorio N.C., Wheeler R.M., Goins G.D., Sanwo-Lewandowski M.M., Mackowiak C.L., Brown C.S., Stutte G.W. (1998). Blue light requirements for crop plants used in bioregenerative life support systems. Life Support Biosph. Sci..

[bib94] Zhang X., He D., Niu G., Yan Z., Song J. (2018). Effects of environment lighting on the growth, photosynthesis, and quality of hydroponic lettuce in a plant factory. Int. J. Agric. Biol. Eng..

[bib95] Zhou Y., Singh B.R. (2002). Red light stimulates flowering and anthocyanin biosynthesis in American cranberry. Plant Growth Regul..

